# Hyperkalemia Risk After Finerenone Initiation in Diabetic Kidney Disease with Elevated Baseline Potassium: A Multicenter Cohort Study

**DOI:** 10.3390/jcm15155758

**Published:** 2026-07-23

**Authors:** Alper Tuna Güven, Serap Yadigar, Murat Özdede, Suat Akgür, Felemez Arslan, Mehmet Sezen, Büşra Özcan, Elif Yıldırım Ayaz, Betül Doğantekin, İlker Atay, Serpil Müge Değer, Mustafa Demir, Sümeyra Koyuncu, Üstün Yılmaz, Ayça İnci, Hülya Çolak, Bülent Demirelli, Serhan Tuğlular, Ebru Gok Oguz, Mehmet Deniz Ayli, Ezgi Avanaz, Fatih Yılmaz, Beyza Doğan, Süleyman Karaköse, Mine Şebnem Karakan, Bahar Gürlek Demirci, Kültigin Türkmen, İsmail Baloğlu, Mehmet Batmazoğlu, Mehmet Mert, Emre Aydın, Fatma Yılmaz Aydın, Ergün Parmaksız, Pınar Özdemir, Bartu Ediz, Zeki Aydın, Mehmet Emin Demir, Siren Sezer, Suat Ünver, Öznur Baykal, Beril Akman, Atila Altuntaş, Mahmud İslam, Sibel Yücel Koçak, Meral Mert, Belda Dursun, Necmi Eren, Simge Bardak Demir, Mukadder Ayşe Bilgiç, Arda Erdut, Gülşah Keskin, Mehmet Horoz, Ahmet Ziya Şahin, Merve Tekinyıldız, Murat Duranay, Serkan Feyyaz Yalın, Hazal Melike Yavuz Umut, Murat Altunok, Ramazan Sarı, Funda Sarı, Dilek Torun, Serdar Kahvecioğlu, Asena Serap Karatutlu, Tülin Akagün, Hüseyin Çelik, Sinan Kazan, İlyas Öztürk, Mehmet Polat, Mehmet Fethullah Aydın, Meral Meşe, Asil Demirezen, Ülver Derici, Eda Altun, Serkan Bakırdöğen, Ekrem Kara, Cüneyt Akgöl, Mahmut Başar Aykent, Ali İlter, Ahmet Ekmekci, Sena Ulu, Mustafa Arıcı, Erkan Şengül, Elif Arı Bakır

**Affiliations:** 1 Division of General Internal Medicine, Department of Internal Medicine, Faculty of Medicine, Marmara University, 34854 İstanbul, Türkiye; 2 Department of Nephrology, Kartal Dr Lütfi Kırdar City Hospital, 34865 İstanbul, Türkiye; 3 Division of General Internal Medicine, Department of Internal Medicine, Faculty of Medicine, Hacettepe University, 06100 Ankara, Türkiye; 4 Nephrology Clinic, Bursa Çekirge State Hospital, 16090 Bursa, Türkiye; 5 Clinic of Internal Medicine, Bakırköy Dr. Sadi Konuk Training and Research Hospital, Health Sciences University, 34147 İstanbul, Türkiye; 6 Department of Nephrology, Bursa City Hospital, 16110 Bursa, Türkiye; 7 Division of Endocrinology and Metabolism, Ataturk Sanatoryum Training and Research Hospital, 06290 Ankara, Türkiye; 8 Department of Internal Medicine, Sultan 2. Abdülhamid Han Training and Research Hospital, Health Sciences University, 34668 İstanbul, Türkiye; 9 Division of Nephrology, Department of Internal Medicine, Faculty of Medicine, Dokuz Eylül University, 35210 İzmir, Türkiye; 10 Nephrology Clinic, Kayseri City Hospital, Health Sciences University, 38080 Kayseri, Türkiye; 11 Nephrology Clinic, Antalya Training and Research Hospital, Health Sciences University, 07100 Antalya, Türkiye; 12 Division of Nephrology, Department of Internal Medicine, Faculty of Medicine, Akdeniz University, 07070 Antalya, Türkiye; 13 Nephrology Clinic, İzmir City Hospital, Health Sciences University, 35540 İzmir, Türkiye; 14 Nephrology Clinic, Marmara University Hospital, 34899 İstanbul, Türkiye; 15 Division of Nephrology, Department of Internal Medicine, Faculty of Medicine, Marmara University, 34722 İstanbul, Türkiye; 16 Department of Nephrology, Etlik City Hospital, Health Sciences University, 06170 Ankara, Türkiye; 17 Nephrology Clinic, Antalya City Hospital, 07080 Antalya, Türkiye; 18 Nephrology Clinic, Antalya City Hospital, Health Sciences University, 07080 Antalya, Türkiye; 19 Nephrology Clinic, Konya City Hospital, Health Sciences University, 42020 Konya, Türkiye; 20 Division of Nephrology, Department of Internal Medicine, Ankara Yıldırım Beyazıt University, 06760 Ankara, Türkiye; 21 Division of Nephrology, Department of Internal Medicine, Faculty of Medicine, Necmettin Erbakan University, 42090 Konya, Türkiye; 22 Nephrology Clinic, Denizli State Hospital, 20010 Denizli, Türkiye; 23 Nephrology Clinic, Özel Sağlık Hospital, 20040 Denizli, Türkiye; 24 Division of Nephrology, Department of Internal Medicine, Faculty of Medicine, Dicle University, 21280 Diyarbakır, Türkiye; 25 Department of Internal Medicine, Faculty of Medicine, Dicle University, 21280 Diyarbakır, Türkiye; 26 Department of Nephrology, Darıca Farabi Training and Research Hospital, Health Sciences University, 41700 Kocaeli, Türkiye; 27 Internal Medicine and Organ Transplantation, Department of Nephrology, Medicana International Ankara Hospital, School of Medicine, Atılım University, 06830 Ankara, Türkiye; 28 Department of Internal Medicine and Nephrology, Acıbadem Hospital, 34758 İstanbul, Türkiye; 29 Department of Nephrology, Basaksehir Cam and Sakura City Hospital, 34480 İstanbul, Türkiye; 30 Department of Internal Medicine and Nephrology, Acıbadem Hospital, 34758 Ankara, Turkey; 31 Division of Nephrology, Department of Internal Medicine, Faculty of Medicine, Suleyman Demirel University, 32260 Isparta, Türkiye; 32 Department of Nephrology, Faculty of Medicine, Sakarya University, 54050 Sakarya, Türkiye; 33 Department of Nephrology, Bakirkoy Dr. Sadi Konuk Training and Research Hospital, Health Sciences University, 34147 İstanbul, Türkiye; 34 Endocrinology Clinic, Bakırköy Dr. Sadi Konuk Training and Research Hospital, Health Sciences University, 34147 İstanbul, Türkiye; 35 Department of Nephrology, Faculty of Medicine, Pamukkale University, 20160 Denizli, Türkiye; 36 Department of Nephrology, Faculty of Medicine, Kocaeli University, 41001 Kocaeli, Türkiye; 37 Department of Nephrology, Yenimahalle Education and Research Hospital, Ankara Yildirim Beyazit University, 06760 Ankara, Türkiye; 38 Department of Nephrology, Ankara Medical Park Hospital, 06120 Ankara, Türkiye; 39 Division of Nephrology, Department of Internal Medicine, Faculty of Medicine, Hacettepe University, 06100 Ankara, Türkiye; 40 Division of Endocrinology and Metabolism, Haydarpaşa Numune Training and Research Hospital, Health Sciences University, 34668 İstanbul, Türkiye; 41 Department of Nephrology, Faculty of Medicine, Health Sciences University Adana, 01370 Adana, Türkiye; 42 Department of Nephrology, Adana City Hospital, Health Sciences University, 01370 Adana, Türkiye; 43 Division of Nephrology, Department of Internal Medicine, Ankara Training and Research Hospital, 06230 Ankara, Türkiye; 44 Department of Nephrology, Trabzon Kanuni Training and Research Hospital, 61250 Trabzon, Türkiye; 45 Division of Endocrinology and Metabolism, Department of Internal Medicine, Faculty of Medicine, Akdeniz University, 07070 Antalya, Türkiye; 46 Department of Internal Medicine, Faculty of Medicine, Akdeniz University, 07070 Antalya, Türkiye; 47 Adana Dr Turgut Noyan Teaching and Research Center, Department of Nephrology, Faculty of Medicine, Baskent University, 06790 Adana, Türkiye; 48 Nephrology Clinic, Bursa Yuksek Ihtisas Training and Research Hospital, Health Sciences University, 16310 Bursa, Türkiye; 49 Nephrology Clinic, Bursa Yuksek Ihtisas Training and Research Hospital, 16310 Bursa, Türkiye; 50 Nephrology Clinic, Faculty of Medicine, Giresun University, 28200 Giresun, Türkiye; 51 Department of Nephrology, Bursa Acıbadem Hospital, 16210 Bursa, Türkiye; 52 Division of Nephrology, Department of Internal Medicine, Afyonkarahisar Health Sciences University, 03030 Afyonkarahisar, Türkiye; 53 Department of Nephrology, Kahramanmaras Necip Fazil City Hospital, 46050 Kahramanmaraş, Türkiye; 54 Karabük Training and Research Hospital, 78200 Karabük, Türkiye; 55 Department of Nephrology, Balıkesir Atatürk City Hospital, 10100 Balıkesir, Türkiye; 56 Department of Nephrology, Faculty of Medicine, Gazi University, 06560 Ankara, Türkiye; 57 Department of Nephrology, Kocaeli City Hospital, 41060 Kocaeli, Türkiye; 58 Division of Nephrology, Department of Internal Medicine, School of Medicine, Canakkale Onsekiz Mart University, 17100 Çanakkale, Türkiye; 59 Division of Nephrology, Department of Internal Medicine, Faculty of Medicine, Recep Tayyip Erdogan University, 53100 Rize, Türkiye; 60 Burdur State Hospital, 15100 Burdur, Türkiye; 61 Department of Nephrology, Şişli Hamidiye Etfal Training and Research Hospital, 34371 İstanbul, Türkiye; 62 Nephrology Clinic, Kütahya Evliya Çelebi Education and Research Hospital, 43040 Kütahya, Türkiye; 63 Department of Cardiology, Faculty of Medicine, Memorial Göztepe Hospital, Bahçeşehir University, 34349 İstanbul, Türkiye; 64 Department of Nephrology, Sultan Abdülhamid Han Training and Research Hospital, Health Sciences University, 34668 İstanbul, Türkiye; 65 Department of Nephrology, Health Sciences University Kocaeli City Hospital, 41060 Kocaeli, Türkiye; dr.erkansengul@hotmail.com; 66 Department of Nephrology, Health Sciences University Kartal Dr Lütfi Kırdar City Hospital, 34865 İstanbul, Türkiye; elifaribakir@gmail.com

**Keywords:** diabetes complications, diabetic nephropathies, hyperkalemia, mineralocorticoid receptor antagonists, retrospective study

## Abstract

**Background/Objectives**: Finerenone improves cardiovascular and renal outcomes in diabetic kidney disease (DKD), but hyperkalemia remains a key safety concern. Patients with elevated baseline potassium levels (≥4.9 mEq/L) are largely excluded from clinical trials, and real-world data in this population are scarce. **Methods**: In this retrospective multicenter cohort study derived from the FINE-TURK cohort, adults with DKD who initiated finerenone with baseline potassium ≥ 4.9 mEq/L were included. The primary outcome was clinically significant hyperkalemia (K ≥ 5.5 mEq/L) within three months. Multivariable logistic regression analyses were used to identify associated factors, with multiple sensitivity analyses performed. **Results**: A total of 166 patients were included, of whom 47 (28.3%) had baseline potassium levels between 5.1 and 5.5 mEq/L. Of the 166 patients, 35 (21.1%) reached the primary outcome, and 10 (6%) patients had follow-up potassium ≥ 6.0 mEq/L. 126 (76.8%) patients required no intervention, 24 (14.6%) were initiated on potassium binders, and finerenone was discontinued in only 12 (7.3%) patients. Lower baseline estimated glomerular filtration rate, baseline urinary albumin, loop diuretic use and 20 mg finerenone dose were associated with the primary outcome. In contrast, baseline potassium was not associated with the primary outcome. **Conclusions**: In patients with DKD and elevated baseline potassium levels, finerenone initiation was associated with manageable rates of hyperkalemia. Our findings support the cautious use of finerenone in selected patients under close monitoring, as well as highlight the need for a multidimensional approach to hyperkalemia risk assessment.

## 1. Introduction

Type 2 diabetes (T2D) and diabetic kidney disease (DKD) represent a major and growing global health burden, contributing substantially to cardiovascular and renal morbidity and mortality, collectively known as cardio–renal–metabolic (CRM) disease. Globally, approximately 40% of individuals with type 2 diabetes develop diabetic kidney disease, making diabetes the leading cause of chronic kidney disease worldwide [1,2,3,4]. Over the past decades, significant therapeutic advances—particularly with renin–angiotensin–aldosterone system (RAAS) inhibitors, including angiotensin-converting enzyme inhibitors (ACEi) and angiotensin receptor blockers (ARBs), as well as sodium–glucose cotransporter-2 (SGLT2) inhibitors—have markedly improved cardiovascular and renal outcomes in this population [5,6,7,8,9]. Despite these therapeutic advances, a considerable residual risk of kidney disease progression and cardiovascular events persists, underscoring the importance of early identification, comprehensive risk assessment, and the development of additional therapies targeting complementary pathophysiological pathways [10].

Due to increased aldosterone activity in CRM diseases, steroidal mineralocorticoid receptor antagonists (MRAs) such as spironolactone and eplerenone play a significant role in the management of heart failure; however, their use has been largely limited by the risk of hyperkalemia in chronic kidney disease (CKD) [11]. Finerenone, a novel nonsteroidal MRA, has demonstrated significant renal and cardiovascular benefits in patients with DKD in large randomized controlled trials [12,13]. Importantly, compared with steroidal MRAs, finerenone has been associated with a more favorable safety profile with respect to hyperkalemia [14,15]. Nevertheless, hyperkalemia remains one of the most clinically relevant adverse effects and continues to influence prescribing decisions, especially in patients with impaired kidney function or elevated baseline potassium levels. The pivotal trials of finerenone, including FIDELIO-DKD and FIGARO-DKD [12,13], largely excluded patients with higher baseline potassium levels (i.e., potassium ≥ 4.9 mEq/L), and current prescribing recommendations on the manufacturer’s label, which are derived from the aforementioned trials, reflect similar criteria [16]. As a result, only selected patients with borderline hyperkalemia (i.e., potassium ≥ 4.9 mEq/L) are initiated on finerenone, and patients with mild hyperkalemia (i.e., potassium ≥ 5.1 mEq/L) are not initiated on finerenone at all. These scenarios are frequently encountered in routine clinical practice, creating a gap between trial-based recommendations and real-world decision-making. Consequently, clinicians frequently face uncertainty when balancing the potential cardiorenal benefits of finerenone against concerns regarding hyperkalemia in patients who would have been excluded from pivotal clinical trials. Meanwhile, prior analyses [14], including our earlier work from the FINE-TURK cohort [17], have identified baseline potassium and estimated glomerular filtration rate (eGFR) as key determinants of hyperkalemia risk in a general DKD population. Whether these associations remain applicable in patients with elevated baseline potassium levels remains uncertain. Therefore, the primary objective of the present study was to evaluate the incidence of clinically significant hyperkalemia following finerenone initiation in patients with DKD and elevated baseline potassium levels. The secondary objectives were to characterize potassium management strategies during follow-up and to identify baseline factors independently associated with clinically significant hyperkalemia.

## 2. Materials and Methods

### 2.1. Study Design and Population

This study was designed as a retrospective cohort analysis derived from the FINE-TURK cohort, a national, multicenter cohort evaluating the real-world use of finerenone in patients with DKD who receive contemporary CRM treatments. The design and overall characteristics of the FINE-TURK cohort have been described previously [18]. For the present analysis, a subgroup of patients from the FINE-TURK cohort was evaluated. Adult patients (≥18 years) with T2D and DKD, with baseline potassium ≥ 4.9 mEq/L, who initiated finerenone, were eligible. Patients were eligible if baseline potassium, baseline estimated glomerular filtration rate (eGFR, calculated using the CKD-EPI equation), and at least one follow-up potassium measurement within three months of finerenone initiation were available. If more than one potassium measurement was available, the higher potassium value was chosen as the follow-up potassium value to implement a conservative, worst-case approach to hyperkalemia ascertainment.

The present study represents a distinct prespecified subgroup analysis of the FINE-TURK cohort, specifically focusing on patients with elevated baseline potassium levels (≥4.9 mEq/L). This population was not included in our previous FINE-TURK analysis [17], which evaluated finerenone-associated hyperkalemia in patients with baseline potassium ≤ 4.8 mEq/L and more closely reflected the eligibility criteria of pivotal finerenone trials.

### 2.2. Variables of Interest

The following variables were extracted from electronic medical records: age, sex, systolic blood pressure (BP), diastolic BP, body mass index (BMI), clinician-diagnosed or patient-reported comorbidities of hypertension, coronary artery disease (CAD, defined as history of myocardial infarction or revascularization), stroke, and diabetic retinopathy (DRP); finerenone dose initiated (20 mg or 10 mg); current use of any SGLT2 inhibitor, use of any ACE inhibitor or ARB, presence of maximal RAAS inhibition (according to manufacturers’ labels), use of any glucagon-like peptide-1 (GLP-1) receptor agonist, dipeptidyl peptidase-4 (DPP-4) inhibitors, current use of any insulin, metformin, sulfonylurea, calcium channel blocker, beta blocker, alpha blocker, thiazide (or thiazide-like) diuretic, loop diuretic, any antihypertensive, and statin. Regarding laboratory tests, baseline eGFR, baseline serum potassium, baseline urinary albumin (mg/g creatinine), baseline urinary protein (mg/g creatinine), baseline serum sodium, baseline serum albumin, baseline serum uric acid, baseline hemoglobin, and baseline hemoglobin A1c (HbA1c) values were acquired. Baseline potassium levels were dichotomized into 4.9–5.0 mEq/L and 5.1–5.5 mEq/L for further analysis. The former represents the group for borderline hyperkalemia, for whom the manufacturer’s label states finerenone initiation may be considered according to patient characteristics with close potassium follow-up. The latter represents patients with mild hyperkalemia, for whom finerenone is not recommended according to the manufacturer’s prescribing information.

### 2.3. Outcome Definition

The primary outcome was clinically significant hyperkalemia, defined as a follow-up potassium level of ≥5.5 mEq/L, within three months of finerenone initiation. Secondary outcomes included potassium-lowering interventions, bicarbonate therapy, finerenone discontinuation, and the distribution of follow-up potassium categories. The potassium threshold was selected based on the manufacturer’s label and contemporary clinical guidelines, which recommend treatment cessation at potassium levels ≥ 5.5 mEq/L. In addition, follow-up potassium levels were also analyzed as an ordinal variable, categorized as <5.0, 5.0–5.4, 5.5–5.9, and ≥6.0 mEq/L. This approach was used to better characterize the distribution and severity of hyperkalemia in the cohort and to capture clinically relevant gradients of risk beyond a single threshold. Furthermore, hyperkalemia management strategies, including no intervention, initiation of potassium binders, finerenone discontinuation, and initiation of bicarbonate therapy, were evaluated and grouped according to follow-up potassium levels.

### 2.4. Statistical Analysis

Continuous variables were assessed for normality using the Shapiro–Wilk test. No variables were distributed normally, and non-normally distributed variables were presented as medians with interquartile ranges. Between-group comparisons of continuous variables were performed using the Mann–Whitney U test. Categorical variables were shown as counts and percentages, and between-group comparisons were performed using the chi-square test. To evaluate factors associated with clinically significant hyperkalemia, logistic regression (LR) analyses were performed. Univariate LR analyses were initially conducted to assess crude associations between candidate variables and clinically significant hyperkalemia. Covariates included in the multivariable model were selected a priori based on biological plausibility, existing literature, and findings from previous analyses of the FINE-TURK cohort rather than solely on univariate statistical significance. The final model included baseline potassium, baseline eGFR, log-transformed baseline urinary albumin, finerenone dose, and loop diuretic use. Given the limited number of outcome events, the number of covariates included in each multivariable model was restricted to minimize overfitting, in accordance with events-per-variable principles. Baseline potassium was modeled as a continuous variable and scaled per 0.1 mEq/L increase to facilitate clinical interpretation of effect estimates. Baseline urinary albumin and protein were logarithmically transformed because of their right-skewed distributions. Finerenone dose and loop diuretic use were analyzed as binary variables. Results are presented as odds ratios (ORs) with corresponding 95% confidence intervals (CIs). Model calibration was evaluated using the Hosmer–Lemeshow goodness-of-fit test, and model discrimination was assessed by calculating the area under the receiver operating characteristic curve. To evaluate the robustness of the findings, prespecified sensitivity analyses were performed using alternative model specifications, including substitution of baseline urinary albumin with baseline urinary protein, replacement of loop diuretic use with diabetic retinopathy status, replacement of loop diuretic use with thiazide diuretic use, and removing loop diuretics from the model. Observations with missing values for variables included in a given analysis were excluded from that specific analysis (complete-case analysis). A two-sided *p* value < 0.05 was considered statistically significant. All statistical analyses were performed using IBM SPSS Statistics, version 25 (IBM Corp., Armonk, NY, USA).

## 3. Results

### 3.1. Study Population and Baseline Characteristics

A total of 166 patients with DKD and baseline potassium ≥ 4.9 mEq/L were included. The median age was 61.5 (54–70) years, and 51.2% were female. The median baseline potassium level was 5.0 (4.9–5.1) mEq/L. When stratified by baseline potassium categories, patients with mild hyperkalemia had higher BMI (30.9 vs. 28.4, *p* = 0.008) and lower diastolic BP (76 vs. 80, *p* = 0.023) compared to borderline hyperkalemic patients. They also exhibited significantly higher levels of baseline urinary albumin (1900 vs. 949, *p* = 0.018) and baseline urinary protein (2269 vs. 1423, *p* = 0.044). Initiation of finerenone at 20 mg was also more frequent in patients with baseline mild hyperkalemia (12.8% vs. 2.5%, *p* = 0.009), although the absolute number of patients receiving 20 mg was small (6 vs. 3). When patients were stratified according to follow-up potassium levels, clinically significant hyperkalemia occurred in 35 patients (21.1%). Patients who developed clinically significant hyperkalemia had lower baseline eGFR (39 vs. 47, *p* = 0.011) and higher levels of baseline urinary albumin (3096 vs. 903, *p* < 0.001) and baseline urinary protein levels (4260 vs. 1419, *p* = 0.001). DRP was more frequent (66.7% vs. 45.8%, *p* = 0.041), and loop diuretic use was more common in patients who developed clinically significant hyperkalemia (31.4% vs. 12.6%, *p* = 0.008). Table 1 summarizes the baseline characteristics according to baseline potassium categories and follow-up hyperkalemia status in detail.

### 3.2. Potassium Trajectory and Distribution

The median follow-up potassium level was 5.1 (4.9–5.4) mEq/L. Patients with baseline mild hyperkalemia had higher follow-up potassium values compared to borderline hyperkalemic patients (5.2 vs. 5.0 mEq/L, *p* = 0.003). However, the magnitude of potassium change did not differ significantly between baseline potassium groups (0.1 vs. 0.1, *p* = 0.162) (Table 2). When categorized ordinally, 46 (27.7%) patients had follow-up potassium levels < 5.0 mEq/L, 85 (51.2%) had levels of 5.0–5.4 mEq/L, 25 (15.1%) had levels of 5.5–5.9 mEq/L, and 10 (6.0%) had levels ≥ 6.0 mEq/L. Patients with baseline mild hyperkalemia levels approached statistical significance with a shift toward higher follow-up potassium categories (*p* = 0.052). Table 2 summarizes the distribution of follow-up potassium levels according to baseline potassium categories in detail.

### 3.3. Hyperkalemia Management

Data on hyperkalemia management were missing for 2 patients; therefore, hyperkalemia management analysis was conducted on 164 patients. Overall, no intervention was required in 76.8% of patients. Follow-up potassium levels were associated with different intervention strategies (*p* < 0.001). Among patients with follow-up potassium levels of 5.5–5.9 mEq/L, potassium binders were initiated in 65.2%, and finerenone was discontinued only in 13% of patients. However, in patients with follow-up potassium levels ≥ 6.0 mEq/L, finerenone was discontinued in all cases. Bicarbonate therapy was infrequently used and limited to two patients with moderate hyperkalemia. Table 3 summarizes the management strategies and treatment modifications following hyperkalemia in detail.

### 3.4. Factors Associated with Clinically Significant Hyperkalemia

**Figure 1:**
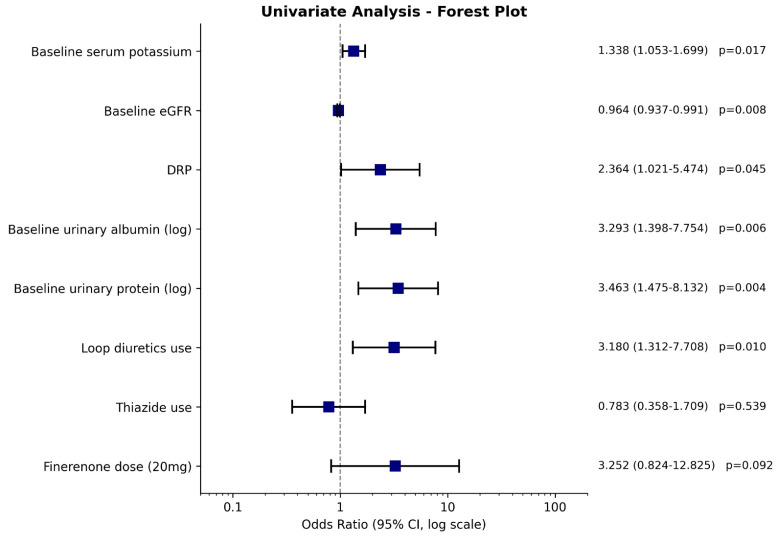
Forest plot of factors associated with clinically significant hyperkalemia in univariate logistic regression analyses. CI: Confidence interval, DRP: Diabetic retinopathy, eGFR: Estimated glomerular filtration rate.

**Figure 2:**
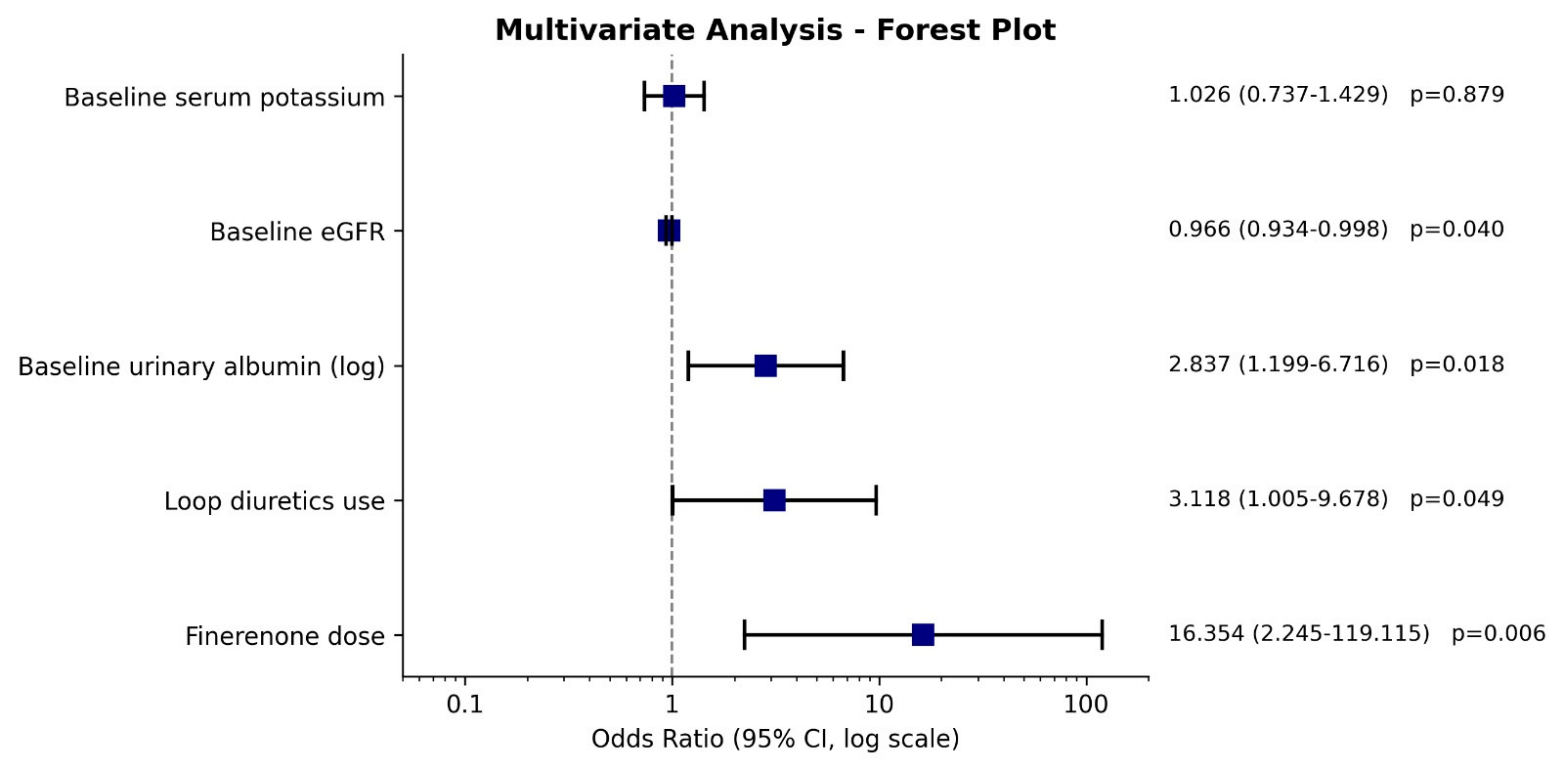
Forest plot of factors associated with clinically significant hyperkalemia in multivariable logistic regression analyses. CI: Confidence interval, eGFR: Estimated glomerular filtration rate.

In univariate LR analysis (Figure 1), baseline potassium, baseline eGFR, DRP, baseline urinary albumin (log-transformed) and protein, and loop diuretic use were associated with clinically significant hyperkalemia. Thiazide use and finerenone dose were not associated with clinically significant hyperkalemia in univariate logistic regression analysis. In multivariable logistic regression analysis (Figure 2), baseline potassium was not independently associated with clinically significant hyperkalemia (OR 1.026, 95% CI 0.737–1.429, *p* = 0.879). Conversely, lower baseline eGFR (OR 0.966, 95% CI 0.934–0.998, *p* = 0.040), higher baseline urinary albumin (log-transformed; OR 2.837, 95% CI 1.199–6.716, *p* = 0.018), loop diuretic use (OR 3.118, 95% CI 1.005–9.678, *p* = 0.049), and initiation with a 20 mg finerenone dose (OR 16.354, 95% CI 2.245–119.115, *p* = 0.006) were independently associated with clinically significant hyperkalemia. The model demonstrated moderate explanatory performance (Nagelkerke R^2^ = 0.274), acceptable goodness-of-fit according to the Hosmer–Lemeshow test (χ^2^ = 11.985, df = 8, *p* = 0.152) and acceptable discrimination, with an area under the receiver operating characteristic curve of 0.790 (95% CI, 0.686–0.894; *p* < 0.001). Table 4 demonstrates the factors associated with hyperkalemia in univariate and multivariable LR analyses in detail.

### 3.5. Sensitivity Analyses

Sensitivity analyses using alternative multivariable model specifications demonstrated overall robustness of the findings (Table 5). Replacement of baseline urinary albumin with baseline urinary protein yielded comparable results, with lower eGFR, higher baseline urinary protein, and 20 mg finerenone dose remaining independently associated with clinically significant hyperkalemia, whereas baseline potassium and loop diuretic use were not associated. Similarly, replacing loop diuretic use with diabetic retinopathy and thiazide diuretic use did not materially alter the results, with baseline urinary albumin and 20 mg finerenone dose remaining significant factors, while baseline potassium, diabetic retinopathy and thiazide diuretic use were not associated with hyperkalemia. In an additional model excluding loop diuretic use, lower baseline eGFR, higher baseline urinary albumin excretion, and 20 mg finerenone dose remained independently associated with clinically significant hyperkalemia. Across all sensitivity analyses, baseline potassium consistently lacked an independent association with clinically significant hyperkalemia, whereas the association between 20 mg finerenone dose and hyperkalemia remained robust.

## 4. Discussion

In this multicenter retrospective cohort analysis derived from the FINE-TURK cohort, we evaluated potassium dynamics, management strategies, and factors associated with clinically significant hyperkalemia in patients with DKD who were initiated on finerenone despite borderline or mild hyperkalemia. This represents a clinically relevant yet significantly understudied population that is largely, if not totally, excluded from randomized trials [12,13,19]. Clinically significant hyperkalemia occurred in approximately one-fifth of patients; however, most cases were mild and manageable, and the majority of patients did not require treatment cessation. Importantly, in contrast to patients with baseline potassium levels ≤ 4.8 mEq/L [14,17], baseline potassium was not independently associated with clinically significant hyperkalemia, whereas baseline urinary albumin demonstrated a more consistent association. These apparently differing observations should not be interpreted as contradictory findings but rather as complementary evidence derived from distinct clinical populations. While our previous FINE-TURK analysis [17] evaluated patients with baseline potassium levels within the conventional finerenone initiation range, the present study specifically focused on patients with elevated baseline potassium levels who were largely excluded from randomized clinical trials. Therefore, the determinants of finerenone-associated hyperkalemia may differ between these two clinically distinct scenarios. To the best of our knowledge, this is the first real-world study specifically evaluating finerenone-associated hyperkalemia in patients with elevated baseline potassium levels—a population largely excluded from clinical trials.

The percentages of patients experiencing potassium levels > 5.5 mEq/L and >6.0 mEq/L were 21.4% and 4.5%, respectively, in the FIDELIO-DKD trial and 13.5% and 2.3%, respectively, in the FIGARO-DKD trial [12,13,14]. However, observational FINE-REAL study and a recent cohort study using the FOUNTAIN platform demonstrated that potassium levels > 5.5 mEq/L were observed in only 8% and 1.2% of the patients, respectively [20,21]. These rates are similar to our group’s recent study [17], in which similar eligibility criteria to landmark trials were used, which demonstrated that potassium levels ≥ 5.5 mEq/L were observed only in 7.3% of the patients. Our current study demonstrated that potassium levels > 5.5 mEq/L and >6.0 mEq/L were almost similar to findings from the FIDELIO-DKD trial [13], indicating a risk profile comparable to that observed in clinical trials. The observed difference in hyperkalemia between clinical trials and observational studies may be explained by exceedingly low SGLT2 inhibitor utilization in landmark trials (4.4% and 8.5% in the FIDELIO-DKD and the FIGARO-DKD, respectively, vs. 38.0% in the FOUNTAIN platform study and 89% in our previous study) and a higher rate of the 10 mg finerenone dose in cohort studies compared to 20 mg dose (85% in both the FOUNTAIN platform study and the FINE-REAL study and 83% in our previous study).

Analyzing the factors associated with clinically significant hyperkalemia yielded several unexpected findings that contrasted with published literature data. Our previous study [17] had demonstrated that baseline potassium, baseline eGFR and finerenone dose were the most prominent factors associated with hyperkalemia, followed by thiazide diuretic use. Similarly, post hoc safety analysis of the FIDELIO-DKD trial, and the FOUNTAIN platform study indicated that baseline potassium, baseline eGFR, diuretic use, SGLT2 inhibitor use, and baseline urinary albumin were associated with hyperkalemia [14,20]. However, baseline potassium and thiazide diuretics were not associated with clinically significant hyperkalemia in the current study, and loop diuretics use was associated with clinically significant hyperkalemia. There may be several explanations for these findings. Firstly, approximately three-quarters of the patients in our study had baseline potassium levels of either 4.9 mEq/L or 5 mEq/L. The limited variability in potassium concentrations may have reduced its discriminatory ability as an independent variable. Therefore, the absence of an independent association between baseline potassium and clinically significant hyperkalemia should be interpreted cautiously and it should not be interpreted as indicating that baseline potassium lacks prognostic value in broader DKD populations. Secondly, association of loop diuretics and clinically significant hyperkalemia may reflect confounding by indication, as loop diuretics are more frequently prescribed in patients with advanced CKD, volume overload, or concomitant heart failure [22]. Therefore, loop diuretic use may act as a surrogate marker of disease severity rather than an independent causal factor, since biological causality would be expected in the reverse direction, namely, association with lower odds of hyperkalemia. Taken together, these findings suggest that the determinants of hyperkalemia are context-dependent and may not be generalizable for each baseline potassium and CKD severity level. This pattern mirrors the contemporary classification of CKD, which integrates both eGFR and albuminuria rather than relying on either parameter alone [22]. Similarly, our findings suggest that finerenone-associated hyperkalemia risk is not driven by a single parameter but rather emerges from a multidimensional interplay between various markers.

From a clinical perspective, our findings provide reassurance regarding the safety profile of finerenone in patients with borderline or mild hyperkalemia. Although rates of drug discontinuation in our cohort were higher (7.3%) than those reported in the landmark trials of FIDELIO-DKD (2.3%) and FIGARO-DKD (1.2%), this likely reflects real-world practice patterns, including more conservative decision-making in response to rising potassium levels, as well as an already higher baseline potassium starting point. It is of particular importance to note that 13.6% of the patients in the FIDELIO-DKD trial had baseline potassium > 4.8 mEq/L, and 6.9% had baseline potassium > 5 mEq/L, similar to our study’s eligibility criteria. Similarly, 4.9% of the patients in the FINE-REAL observational study had baseline potassium > 5.0 mEq/L. However, clinically significant hyperkalemia rates, factors associated with clinically significant rates, and management strategies including drug discontinuation were not reported in these specific subgroups [14,21]. Lastly, the observed incidence of clinically significant hyperkalemia should not be interpreted as trivial. Rather, these findings indicate that finerenone initiation in patients with elevated baseline potassium requires careful patient selection and structured potassium monitoring, while also demonstrating that most events can be managed without permanent treatment discontinuation.

Since our study evaluated potassium dynamics within the first three months of finerenone initiation, potassium elevations beyond this period may seem like a major limitation. The FIDELIO-DKD study demonstrated that finerenone elevated potassium levels by 0.23 mEq/L at month 4, and the levels remained largely stable thereafter [13]. The FIGARO-DKD study, which included DKD patients with lower disease severity, demonstrated that finerenone elevated potassium levels by 0.16 mEq/L at month 1, and potassium levels remained largely stable thereafter [12]. Moreover, a recent cohort study demonstrated that mean potassium increased by 0.1 mEq/L at 4 months of finerenone initiation and remained unchanged at 12 months (an increase of 0.1 mEq/L from baseline) [20]. Clinical trials, as well as the latter cohort data, provide assurance for our study with regard to its ability to capture clinically significant hyperkalemia, since potassium levels are expected to elevate within the first months of finerenone initiation and become stable thereafter.

Another clinically relevant finding of our study was the independent association between 20 mg finerenone dose and clinically significant hyperkalemia. Although the estimated effect size was accompanied by wide confidence intervals, reflecting the limited number of patients who started on 20 mg finerenone, this association remained directionally consistent across all sensitivity analyses. Therefore, while the precise magnitude of risk should be interpreted cautiously, our findings support a potential dose-dependent relationship between finerenone exposure and hyperkalemia risk. This observation is biologically plausible and consistent with the dose-dependent increases in potassium observed in our prior analyses from the FINE-TURK cohort [17].

Unlike previous studies evaluating broader diabetic kidney disease (DKD) populations, the present study specifically focused on patients with elevated baseline potassium levels, representing a clinically challenging population that has been largely excluded from the pivotal finerenone trials. Accordingly, our findings should be interpreted as complementary to, rather than contradictory to, the evidence derived from randomized clinical trials. Whereas the pivotal finerenone trials established the efficacy and safety of finerenone in carefully selected patients, our real-world analysis extends these observations to a higher-risk population commonly encountered in routine clinical practice, thereby helping to bridge the gap between trial eligibility and real-world treatment decisions. Importantly, because severe hyperkalemia may result in clinically significant electrocardiographic abnormalities and life-threatening arrhythmias, accurate identification of patients at increased risk remains essential [23]. Collectively, our findings suggest that elevated baseline potassium should not automatically preclude finerenone initiation. Instead, treatment decisions should be individualized by integrating kidney function, albuminuria, concomitant diuretic therapy, and availability of close potassium monitoring, rather than relying on baseline potassium concentration alone. Future prospective studies with standardized potassium monitoring protocols, longer follow-up, and larger numbers of patients receiving higher finerenone doses are warranted to validate these findings and further refine individualized risk prediction strategies.

## 5. Strengths and Limitations

Firstly, the retrospective observational design precludes causal inference and the findings remain susceptible to residual confounding. In addition, because finerenone initiation in patients with elevated baseline potassium was based on physician discretion rather than standardized treatment criteria, selection bias and confounding by indication cannot be excluded. Clinicians may have preferentially selected patients whom they considered less likely to develop severe hyperkalemia despite elevated baseline potassium levels; therefore, the present cohort may represent a selected subgroup rather than the broader population of patients with DKD and elevated baseline potassium encountered in routine clinical practice. Furthermore, treatment initiation, finerenone dose selection, potassium monitoring, and hyperkalemia management were not standardized across participating centers, reflecting real-world practice but introducing additional variability in patient management. Similarly, patients who underwent more frequent potassium monitoring may have had a greater likelihood of hyperkalemia detection, resulting in surveillance bias. Secondly, the sample size, particularly the number of clinically significant hyperkalemia events, was relatively limited, which may have reduced statistical power and contributed to imprecision in some estimates, as reflected by the wide confidence intervals for certain variables. Thirdly, the timing and frequency of potassium measurements were not standardized and could not be comprehensively evaluated in this retrospective study. Consequently, patients who underwent more frequent potassium monitoring may have had a greater likelihood of hyperkalemia detection, introducing surveillance bias. Furthermore, the use of the highest available potassium value during follow-up was intentionally chosen to provide a conservative estimate of clinically significant hyperkalemia and to minimize the risk of underestimating clinically relevant events. Finally, although initiation with the 20 mg dose was independently associated with clinically significant hyperkalemia, this finding should be interpreted cautiously because only a small number of patients received this dose, resulting in a wide confidence interval and limited precision of the effect estimate. Consequently, this observation should be considered hypothesis-generating rather than definitive and warrants confirmation in larger prospective cohorts.

Despite these limitations, the present study has several important strengths. To our knowledge, it is the first real-world study specifically evaluating finerenone-associated hyperkalemia in patients with elevated baseline potassium levels, a clinically challenging population which has been largely excluded from pivotal randomized trials. Furthermore, the multicenter design enhances the generalizability of the findings, while the use of predefined multivariable models and multiple sensitivity analyses supports the robustness and consistency of the observed associations.

## 6. Conclusions

In this multicenter real-world cohort, finerenone-associated clinically significant hyperkalemia in patients with diabetic kidney disease and elevated baseline potassium levels occurred at rates comparable to those reported in pivotal clinical trials and was generally manageable without treatment discontinuation. Baseline potassium was not independently associated with hyperkalemia risk, whereas reduced kidney function, greater albuminuria and higher finerenone dose emerged as more clinically relevant predictors. These findings suggest that baseline potassium should not be considered in isolation when evaluating eligibility for finerenone initiation; instead, treatment decisions should incorporate overall kidney disease severity and individualized clinical risk, together with appropriate potassium monitoring. Also, our findings support the cautious initiation of finerenone in carefully selected patients with borderline or mildly elevated baseline potassium levels, provided that structured potassium monitoring and appropriate mitigation strategies are available. Future prospective studies with standardized monitoring protocols and longer follow-up are required to validate these observations and further refine individualized risk stratification.

## Figures and Tables

**Table 1:** Baseline patient characteristics according to baseline potassium categories and follow-up hyperkalemia status.

	Total	Baseline Potassium			Follow-up Potassium		
	(N = 166)	K = 4.9–5.0 (N = 119)	K = 5.1–5.5 (N = 47)	*p* *	K < 5.5 (N = 131)	K ≥ 5.5 (N = 35)	*p* *
Demographics and Physical Examination							
Age	61.5 (54–70)	61 (54–70)	62 (54–69)	0.87	62 (54–69)	59 (54–71)	0.804
Sex				0.312			0.681
Female	85 (51.2%)	58 (48.7%)	27 (57.4%)		66 (50.4%)	19 (54.3%)	
Male	81 (48.8%)	61 (51.3%)	20 (42.6%)		65 (49.6%)	16 (45.7%)	
BMI	29.1 (26.3–33.0)	28.4 (25.7–32.7)	30.9 (28–33.3)	**0.008**	29.0 (25.8–33)	29.4 (26.9–33.1)	0.664
Systolic BP	130 (125–144)	130 (127–140)	132 (123–149)	0.786	130 (125–140)	140 (130–154)	0.096
Diastolic BP	80 (72–88)	80 (75–90)	76 (70–80)	**0.023**	80 (70–88)	80 (73–89)	0.575
Comorbidities							
Hypertension	155 (93.4%)	111 (93.3%)	44 (93.6%)	0.937	120 (91.6%)	35 (100%)	0.076
CAD	61 (37.2%)	45 (38.5%)	16 (34%)	0.597	50 (38.8%)	11 (31.4%)	0.426
Stroke	19 (11.7%)	14 (12.1%)	5 (10.6%)	0.797	15 (11.6%)	4 (11.8%)	0.982
DRP	75 (50%)	52 (48.1%)	23 (54.8%)	0.467	55 (45.8%)	20 (66.7%)	**0.041**
RAS Inhibitors							
ACE inhibitor	65 (39.4%)	46 (38.7%)	19 (41.3%)	0.755	51 (39.2%)	14 (40%)	0.934
ARB	80 (48.5%)	56 (47.1%)	24 (52.2%)	0.556	64 (49.2%)	16 (45.7%)	0.712
Maximal RAAS blockade	105 (67.3%)	78 (69%)	27 (62.8%)	0.458	82 (66.7%)	23 (69.7%)	0.742
SGLT-2 Inhibitors and GLP-1 Receptor Agonists							
SGLT-2 inhibitor	142 (85.5%)	100 (84%)	42 (89.4%)	0.379	114 (87%)	28 (80%)	0.294
GLP-1 receptor agonist	1 (0.6%)	0	1 (2.1%)	0.283	1 (0.8%)	0	1
Finerenone							
Finerenone dose				**0.009**			0.095
10 mg	157 (94.6%)	116 (97.5%)	41(87.2%)		126 (96.2%)	31 (88.6%)	
20 mg	9 (5.4%)	3 (2.5%)	6 (12.8%)		5 (3.8%)	4 (11.4%)	
Other Glucose-Lowering Medications							
DPP-4 inhibitor	99 (59.6%)	70 (58.8%)	29 (61.7%)	0.733	79 (60.3%)	20 (57.1%)	0.735
Insulin	90 (56.3%)	61 (53%)	29 (64.4%)	0.191	70 (56%)	20 (57.1%)	0.904
Metformin	70 (42.2%)	51 (42.9%)	19 (40.4%)	0.775	57 (43.5%)	13 (37.1%)	0.498
Sulfonylurea	8 (4.8%)	5 (4.2%)	3 (6.4%)	0.554	5 (3.8%)	3 (8.6%)	0.367
Anti-Hypertensive Medications							
CCB	84 (50.9%)	59 (49.6%)	25 (54.3%)	0.583	61 (46.9%)	23 (65.7%)	**0.048**
Beta-blocker	75 (45.5%)	48 (40.3%)	27 (58.7%)	**0.034**	58 (44.6%)	17 (48.6%)	0.677
Alpha-blocker	20 (12.1%)	13 (10.9%)	7 (15.2%)	0.449	14 (10.8%)	6 (17.1%)	0.305
Thiazide	64 (38.8%)	41 (34.5%)	23 (50%)	0.066	52 (40%)	12 (34.3%)	0.538
Loop diuretics	27 (16.7%)	17 (14.3%)	10 (23.3%)	0.176	16 (12.6%)	11 (31.4%)	**0.008**
Any anti-hypertensive	160 (96.4%)	114 (95.8%)	46 (97.9%)	0.519	125 (95.4%)	35 (100%)	0.197
Lipid-Lowering Medications							
Statin	100 (60.6%)	70 (59.3%)	30 (63.8%)	0.593	80 (61.1%)	20 (58.8%)	0.811
Laboratory							
Baseline potassium (mEq/L)	5.0 (4.9–5.1)	4.9 (4.9–5.0)	5.2 (5.1–5.3)	**<0.001**	4.9 (4.9–5.1)	5.0 (5.0–5.1)	**0.007**
Baseline eGFR (mL/min/1.73m^2^)	44 (34–57)	45 (36–58)	41 (32–55)	0.128	47 (36–60)	39 (32–46)	**0.011**
Baseline urinary albumin (mg/g creatinine)	1392 (373–2901)	949 (247–2370)	1900 (805–4349)	**0.018**	903 (258–2105)	3096 (1527–4808)	**<0.001**
Baseline urinary protein (mg/g creatinine)	1950 (732–4194)	1423 (610–3400)	2269 (1192–5779)	**0.044**	1419 (660–3142)	4260 (1220–6717)	**0.001**
Baseline serum sodium (mEq/L)	139 (137–141)	139 (138–141)	139 (137–141)	0.947	139 (138–141)	138 (137–142)	0.833
Baseline serum albumin (g/dL)	4.1 (3.9–4.3)	4.1 (3.9–4.3)	4.1 (3.8–4.5)	0.619	4.1 (3.9–4.3)	4.0 (3.5–4.3)	0.356
Baseline serum uric acid (mg/dL)	6.4 (5.5–7.5)	6.5 (5.6–7.5)	6.4 (5.2–7.4)	0.564	6.4 (5.3–7.4)	6.7 (5.6–7.6)	0.430
Baseline HbA1c (%)	7.6 (6.7–8.4)	7.4 (6.6–8.4)	7.9 (7–8.5)	0.138	7.6 (6.7–8.4)	7.4 (6.6–8.4)	0.776

ACE: Angiotensin-converting enzyme, ARB: Angiotensin receptor blocker, BMI: Body mass index, BP: Blood pressure, CAD: Coronary artery disease, CCB: Calcium channel blocker, DPP-4: Dipeptidyl peptidase-4, DRP: Diabetic retinopathy, eGFR: Estimated glomerular filtration rate, GLP-1: Glucagon-like peptide-1, HbA1c: Glycated hemoglobin, K: Potassium, RAAS: Renin–angiotensin–aldosterone system, SGLT-2: Sodium-glucose transporter-2. * *p* values with statistical significance are shown in bold.

**Table 2:** Distribution of follow-up potassium levels according to baseline potassium categories.

	Total	Baseline Potassium		
	(N = 166)	K = 4.9–5.0 (N = 119)	K = 5.1–5.5 (N = 47)	*p* *
Follow-up potassium (mEq/L)	5.1 (4.9–5.4)	5 (4.9–5.3)	5.2 (5.1–5.5)	**0.003**
Potassium change	0.1 (0–0.4)	0.1 (0–0.4)	0.1 (-0.1–0.2)	0.162
Follow-up potassium (Ordinal)				**0.052**
<5.0 (mEq/L)	46 (27.7%)	40 (33.6%)	6 (12.8%)	
5.0–5.4 (mEq/L)	85 (51.2%)	58 (48.7%)	28 (59.6%)	
5.5–5.9 (mEq/L)	25 (15.1%)	15 (12.6%)	9 (19.1%)	
≥6.0 (mEq/L)	10 (6%)	6 (5%)	4 (8.5%)	

K: Potassium. * *p* values with statistical significance are shown in bold.

**Table 3:** Management strategies and treatment modifications following hyperkalemia.

	Total	Follow-Up Potassium			
Management for Follow-Up Potassium Levels	(N = 164 *)	<5.0 (mEq/L) (N = 46)	5.0–5.4 (mEq/L) (N = 86)	5.5–5.9 (mEq/L) (N = 23)	≥6.0 (mEq/L) (N = 9)
No intervention	126 (76.8%)	46 (100%)	77 (89.5%)	3 (13%)	0
Potassium binder initiation	24 (14.6%)	0	9 (10.5%)	15 (65.2%)	0
Finerenone cessation	12 (7.3%)	0	0	3 (13%)	9 (100%)
Bicarbonate initiation	2 (1.2%)	0	0	2 (8.7%)	0

* 2 patients’ data on hyperkalemia management were missing.

**Table 4:** Factors associated with hyperkalemia: Univariate and multivariable logistic regression analyses.

**Univariate Analysis**		
	**OR (95%CI)**	***p*** *
Baseline potassium	1.338 (1.053–1.699)	**0.017**
Baseline eGFR	0.964 (0.937–0.991)	**0.008**
DRP	2.364 (1.021–5.474)	**0.045**
Baseline urinary albumin (log-transformed)	3.293 (1.398–7.754)	**0.006**
Baseline urinary protein (log-transformed)	3.463 (1.475–8.132)	**0.004**
Loop diuretics use	3.180 (1.312–7.708)	**0.010**
Thiazide use	0.783 (0.358–1.709)	0.539
Finerenone dose (20 mg)	3.252 (0.824–12.825)	0.092
**Multivariate Analysis**		
	**OR (95%CI)**	***p*** *
Baseline potassium	1.026 (0.737–1.429)	0.879
Baseline eGFR	0.966 (0.934–0.998)	**0.040**
Baseline urinary albumin (log-transformed)	2.837 (1.199–6.716)	**0.018**
Loop diuretics use	3.118 (1.005–9.678)	**0.049**
Finerenone dose	16.354 (2.245–119.115)	**0.006**
Nagelkerke R^2^ = 0.274, Hosmer–Lemeshow goodness-of-fit: χ^2^ = 11.985, df = 8, *p* = 0.152. AUC = 0.790 (95% CI, 0.686–0.894).		

AUC: Area under the receiver operating characteristic curve; CI: Confidence interval; DRP: Diabetic retinopathy; eGFR: Estimated glomerular filtration rate; OR: Odds ratio. * *p* values with statistical significance are shown in bold.

**Table 5:** Sensitivity analyses of multivariable models for factors associated with hyperkalemia.

**Model 1**	**OR (95%CI)**	***p*** *
Baseline potassium	1.064 (0.795–1.425)	0.677
Baseline eGFR	0.960 (0.931–0.990)	**0.010**
Baseline urinary protein (log-transformed)	3.474 (1.339–9.014)	**0.010**
Loop diuretics use	1.712 (0.614–4.770)	0.304
Finerenone dose	17.773 (2.358–133.964)	**0.005**
Nagelkerke R^2^ = 0.248		
**Model 2**	**OR (95%CI)**	***p*** *
Baseline potassium	1.049 (0.754–1.459)	0.779
Baseline eGFR	0.974 (0.943–1.006)	0.114
Baseline urinary albumin (log-transformed)	3.252 (1.202–8.797)	**0.020**
DRP	1.414 (0.487–4.102)	0.524
Finerenone dose	15.630 (1.157–211.206)	**0.038**
Nagelkerke R^2^ = 0.183		
**Model 3**	**OR (95%CI)**	***p*** *
Baseline potassium	1.066 (0.774–1.468)	0.695
Baseline eGFR	0.961 (0.930–0.992)	**0.015**
Baseline urinary albumin (log-transformed)	3.302 (1.353–8.058)	**0.009**
Finerenone dose	15.437 (2.116–112.630)	**0.007**
Nagelkerke R^2^ = 0.240		
**Model 4**	**OR (95%CI)**	***p*** *
Baseline potassium	1.086 (0.784–1.503)	0.619
Baseline eGFR	0.960 (0.930–0.992)	**0.013**
Baseline urinary albumin (log-transformed)	3.322 (1.355–8.142)	**0.009**
Thiazide diuretics use	0.692 (0.254–1.885)	0.471
Finerenone dose	14.103 (1.910–104.159)	**0.009**
Nagelkerke R^2^ = 0.246		

CI: Confidence interval; DRP: Diabetic retinopathy; eGFR: Estimated glomerular filtration rate; OR: Odds ratio. * *p* values with statistical significance are shown in bold.

## Data Availability

SPSS outputs are uploaded in a data repository and are freely available at https://doi.org/10.6084/m9.figshare.32114377. Raw data are available upon reasonable request.
